# No Differential Effects of Two Different Alpha-Band Electrical Stimulation Protocols Over Fronto-Parietal Regions on Spatial Attention

**DOI:** 10.3389/fnins.2018.00433

**Published:** 2018-07-03

**Authors:** Martine R. van Schouwenburg, Lynn K. A. Sörensen, Raza de Klerk, Leon C. Reteig, Heleen A. Slagter

**Affiliations:** ^1^Department of Psychology, University of Amsterdam, Amsterdam, Netherlands; ^2^Amsterdam Brain and Cognition, University of Amsterdam, Amsterdam, Netherlands

**Keywords:** alpha oscillations, coherence, connectivity, transcranial alternating current stimulation, visual attention, EEG

## Abstract

In a previous study using transcranial alternating current stimulation (tACS), we found preliminary evidence that phase coherence in the alpha band (8–12 Hz) within the fronto-parietal network may critically support top-down control of spatial attention (van Schouwenburg et al., 2017). Specifically, synchronous alpha-band stimulation over the right frontal and parietal cortex (0° relative phase) was associated with changes in performance and fronto-parietal coherence during a spatial attention task as compared to sham stimulation. In the current study, we firstly aimed to replicate these findings with synchronous tACS. Second, we extended our previous protocol by adding a second tACS condition in which the right frontal and parietal cortex were stimulated in a desynchronous fashion (180° relative phase), to test the specificity of the changes observed in our previous study. Participants (*n* = 23) were tested in three different sessions in which they received either synchronous, desynchronous, or sham stimulation over the right frontal and parietal cortex. In contrast to our previous study, we found no spatially selective effects of stimulation on behavior or coherence in either stimulation protocol compared to sham. We highlight some of the differences in study design that may have contributed to this discrepancy in findings and more generally may determine the effectiveness of tACS.

## Introduction

In daily life, our brain is constantly bombarded with more sensory information than it can fully process. This necessitates the selection of sensory information based on goal-relevance, a process subserved by top–down attention. Spatial attention paradigms are widely used to study the neural underpinnings of top–down attention. In these tasks, participants receive a cue that indicates the most likely location of an upcoming target. It has been replicated many times that participants respond faster and more accurately to cued (i.e., attended) targets compared to uncued (i.e., unattended) targets. This benefit has been attributed to the fact that participants can direct their focus of attention in advance to the relevant location (Posner, 1980).

Previous neuroimaging studies have associated a fronto-parietal network centered on the frontal eye fields (FEF) and intraparietal sulcus (IPS) with top-down orienting of attention (Corbetta and Shulman, 2002; Noudoost et al., 2010). Brain stimulation studies confirmed that both the frontal (Moore and Fallah, 2001; Duecker and Sack, 2014; Marshall et al., 2015) and parietal cortex (Cutrell and Marrocco, 2002; Capotosto et al., 2012) are causally involved in this process.

Research has furthermore shown that spatial attention can prioritize processing of relevant over irrelevant or distracting information by modulating the excitability of sensory regions in preparation of the upcoming target, as reflected in the amplitude of alpha-band oscillations (8–12 Hz). Specifically, when attention is cued toward one hemifield, alpha power increases over the hemisphere ipsilateral to the cued location, which processes the unattended hemifield, and decreases over the hemisphere contralateral to the cued location, which processes the attended hemifield (Worden et al., 2000; Sauseng et al., 2005). This finding has led to the hypothesis that alpha oscillations gate attention through inhibition of irrelevant information (for reviews see: Klimesch et al., 2007; Jensen and Mazaheri, 2010).

Occipital alpha oscillations are likely under the control of the fronto-parietal network. Indeed, transcranial magnetic stimulation (TMS) over the frontal or parietal cortex can modulate occipital alpha activity (Capotosto et al., 2009; Sauseng et al., 2011; Marshall et al., 2015). However, exactly how the fronto-parietal network controls occipital alpha activity is unclear.

In a recent study, we tested the hypothesis that the fronto-parietal network employs top–down control over visual regions through communication in the alpha band (van Schouwenburg et al., 2017). Current theorizing suggests that brain regions communicate through neural coherence, such that when activity in two brain regions oscillates in phase, communication is increased, while communication is hampered when activity in brain regions oscillates out of phase (Womelsdorf and Fries, 2007; Sauseng and Klimesch, 2008). In line with this notion, several empiral studies provide correlational evidence suggesting that the fronto-parietal attention network communicates through coherence in the alpha band (Sauseng et al., 2005; Zanto et al., 2010; Sadaghiani et al., 2012; Doesburg et al., 2016). These studies show for example that alpha coherence between frontal cortex and parietal(-occipital) cortex is modulated as a function of attention.

In our recent study, we aimed to provide *causal* evidence for this hypothesis by actively manipulating alpha-band coherence within the fronto-parietal network using transcranial alternating current stimulation (tACS), while participants performed a spatial attention task (van Schouwenburg et al., 2017). Specifically, we placed stimulation electrodes over the frontal and parietal cortex and stimulated these two regions synchronously (i.e., 0° phase difference), which presumably entrains oscillatory activity at the stimulated frequency, and thereby increases coherence between the two stimulated brain regions (Polanía et al., 2012). We found that a group of participants that received sham stimulation showed a spatial bias both in terms of behavior and fronto-parietal connectivity. Behavorially, they responded faster for targets that appeared in the right hemifield and, neurally, right frontal cortex showed stronger connectivity with the contralateral parietal cortex than with the ipsilateral parietal cortex. Interestingly, these spatial biases were absent in a group of participants that received synchronous alpha stimulation over frontal and parietal cortex (van Schouwenburg et al., 2017). These results provide preliminary evidence that alpha coherence in the fronto-parietal network might play an essential role in top–down control of spatial attention.

Here, we aimed to replicate and extend these preliminary findings by improving the design of the study in two important ways. First, given that in our previous study we only compared synchronous stimulation of the frontal and parietal cortex with sham stimulation, our results could potentially be a result of stimulation of either region alone. Therefore, to be able to conclude that the effects were due to the fact that these two regions were stimulated synchronously, they should be compared to a control condition in which the frontal and parietal cortex are also stimulated but not in a synchronous manner. We decided to add a control condition in which the two regions were stimulated in a desynchronous fashion (i.e., 180° phase difference), conform Polanía et al. (2012). As opposed to the synchronous stimulation, this could potentially lead to a decrease in coherence between the two regions. In the current study, we thus compared synchronous stimulation against desynchronous stimulation (and sham). However, a recent modeling study (Saturnino et al., 2017) suggests that these two stimulation conditions might differ not only in terms of relative phase, but also in terms of peak field strength and focality, making the desynchronous stimulation condition a less than ideal control condition (see Discussion).

Second, we used a different version of the spatial attention task that included invalid cues. That is, on 25% of trials, the target appeared in the hemifield *opposite* to the cued hemifield instead of the cued hemifield. On these invalid trials, participants have to redirect their attention upon target presentation in a bottom-up fashion. The addition of this task condition thus allows us to look if the effects of stimulation extend to bottom-up attention or are selective for top-down attention. Moreover, as the design also included neutral cues, which did not provide any spatial information, this also permitted us to separate facilitatory and inhibitory effects of attention at the behavioral and neural level.

Based on our previous study, we predicted that in the sham stimulation condition, participants would display a rightward spatial attention bias (i.e., faster reaction times (RT) on targets in the right hemifield), and that this bias would be reduced by synchronous stimulation. Given our prediction that the desynchronous stimulation would have the opposite effect of synchronous stimulation (a decrease vs. an increase in fronto-parietal coherence), we predicted that the rightward spatial attention bias would be *increased* by desynchronous stimulation. Neurally, we predicted, like in our previous study, a baseline hemispheric bias in terms of fronto-parietal connectivity that is reduced by synchronous stimulation. The opposite effect was expected after desynchronous stimulation (i.e., an increased hemispheric bias). We also examined effects of stimulation on pre-stimulus posterior alpha power. In our previous study, we found hemispheric-selective effects of stimulation on prestimulus alpha asymmetry that were trending toward significance. We anticipate that the implementation of a within-subject design in the current study, compared to a between-subject design previously, might allow us to pick up these potentionally more subtle effects of stimulation because it better takes into account individual differences in baseline activity.

## Materials and Methods

### Participants

Twenty-three healthy participants were tested in a randomized, within-subject design. All participants had normal or corrected-to-normal vision and were right-handed. They were selected based on the following criteria; no (family history of) epilepsy or history of an epileptic seizure, no neurological or psychiatric disorders, no (history of) stroke or other forms of brain damage, no history of a severe concussion, no (history of) meningitis, no use of psychoactive substances, no spondylosis, no scoliosis, no arthritis, no cardiac pacemakers or other implanted medical devices, no metal anywhere in the head, not pregnant, no recent history of fainting or panic attacks, no frequent occurrences of dizziness or headaches, no skin abnormalities such as eczema, no eye conditions. In addition, we instructed participants to abstain from alcohol, non-prescriptive medication and illicit substances 24 h prior to the experiment.

Three participants were excluded because they systematically broke fixation (see EEG data acquisition and analysis). The final sample included 20 participants, 3 of whom were male (mean age 20.6, SD 3.5, range 18–34). The study was carried out in accordance with the recommendations of the ethical committee of the University of Amsterdam. The protocol was approved by the ethical committee of the University of Amsterdam. All participants gave written informed consent in accordance with the Declaration of Helsinki and were paid €10 per hour or participated for research credit.

### Spatial Attention Paradigm

**FIGURE 1:**
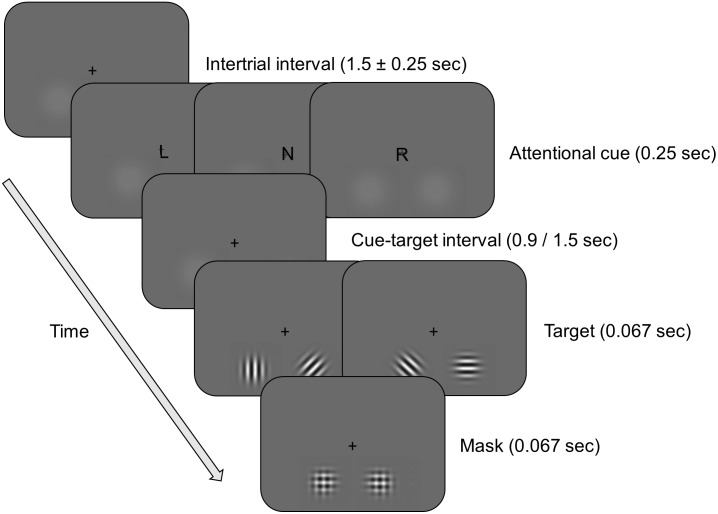
Schematic overview of the spatial attention task. On each trial, participants were presented with a cue, and after a 900 or 1500 ms interval, with a target. The cue indicated that the target was more likely to appear in the left (L) or right (R) hemifield (attention condition, 75% of trials), or contained no information about the likely location of the target (N) (neutral condition, 25% of trials). After a variable delay interval, a target (a horizontally or vertically oriented Gabor patch) and a distractor (a diagonally oriented Gabor patch) stimulus were briefly shown simultaneously in opposite hemifields. Participants had to report the orientation of the target stimulus with a button press. On 75% of the trials in the attention condition the target appeared at the cued location (valid condition), while on the remaining trials the target appeared at the uncued location (invalid condition). The target and distractor were followed by a mask.

Participants were seated in a dark room at a viewing distance of approximately 90 cm from a 24-inch BenQ XL 2420Z monitor. A covert spatial attention task was presented at 144 Hz using PsychToolbox in Matlab (**Figure 1**). The task was adapted from Gould et al. (2011), and differed in several aspects from the task used in our previous study (van Schouwenburg et al., 2017) (for more details, see Discussion). At the beginning of each trial, participants were presented with an attention cue (75% of trials) that indicated whether the upcoming target was more likely to appear in the lower left or lower right quadrant of the screen, or a neutral cue (25% of trials) that contained no spatial information about the upcoming target. The attention cue was valid on 75% of the trials (valid condition). On the remaining trials the target appeared at the uncued location (invalid condition). The neutral cue was followed by a target at the lower left or right location with equal probability. Target stimuli were presented after a cue-target interval of either 900 or 1500 ms, and were paired with distractor stimuli in the opposite hemifield. Target and distractor stimuli both consisted of Gabor patches (spatial frequency: 2.5 cycles per degree, spatial constant of the Gaussian envelope: 0.457°) that only differed in their orientation. The target stimulus was presented horizontally or vertically and participants had to indicate its orientation with a button press (left and right index fingers for horizontal orientation and left and right middle fingers for vertical orientation). For distractor stimuli the pattern was tilted 45° clockwise or counter-clockwise. Distractor and target stimuli were masked with checkerboards of the same size as the Gabor patches. Throughout the experiment two luminance pedestals were presented in the lower left and lower right quadrants of the screen. The pedestals were presented 4.8° of visual angle below the horizontal meridian, and 3.2° to the left and right of the vertical meridian and indicated the location of target and distractor stimuli. Participants were instructed to keep their eyes on the fixation cross for the duration of the experiment and to covertly attend to the cued location. Also, participants were instructed to respond to the targets as fast and accurately as possible.

### Procedure

Participants were tested in three different sessions in which they received either synchronous, desynchronous, or sham stimulation. The order of the different stimulation conditions was randomized across participants and sessions were separated by at least 7 days to prevent carry-over effects (average 7.2 days, range 7–11 days).

In each session, participants were first asked to fill out a visual analog scale to assess subjective feelings prior to stimulation (Bond and Lader, 1974). Next, the stimulation electrodes and EEG were set-up, and participants went through three different stages of the task before the main task began. First, participants performed a practice block of 300 valid trials to get acquainted with the task. During this block they received auditory feedback after each trial. It was ensured that participants would at least achieve a performance of 70% accuracy on the last practice block before they could continue. In the next practice block, the contrast of the target (and distractor) stimuli was titrated to the participants’ individual performance through an adaptive threshold procedure. Again, only trials in the valid condition were presented, and over the course of 200 trials the contrast was adapted to converge on 70% accuracy (see Marshall et al., 2015 for a similar approach). This procedure ensured that participants all started at the same accuracy level with each stimulation condition and also allowed us to detect both beneficial as well as detrimental effects of stimulation on performance. The average presented contrast did not differ between the stimulation conditions [*F*(2,38) = 0.833, *p* = 0.443; mean ± standard deviation: sham 0.15 ± 0.13; synchronous 0.21 ± 0.19; desynchronous 0.22 ± 0.17]. Note that no trial-wise feedback was given during the thresholding procedure to match the conditions of the actual task as closely as possible.

The thresholded stimuli were then used in a third practice block (240 trials), that included neutral and invalid trial conditions. This practice block matched the main task, except that auditory feedback was presented during each practice trial.

Participants then started with the main experiment, which consisted of eight blocks (100 trials, ∼ 5 min/per block) in which participants received alternating blocks of real stimulation and/or sham stimulation (see transcranial alternating current stimulation) (cf. van Schouwenburg et al., 2017). After each completed block, participants received feedback about their performance, and after four blocks, participants had a 15-min break.

After the task was finished, we asked participants again to fill out the visual analog scales and in addition to report possible side effects on a scale from 1 (=not present) to 5 (=extremely noticeable). Side effects included were: headache, neck pain, nausea, muscle contractions in face or neck, tingling or itching sensation, burning sensation, sleepiness, mood changes, and uncomfortable feeling.

### Transcranial Alternating Current Stimulation

**FIGURE 2:**
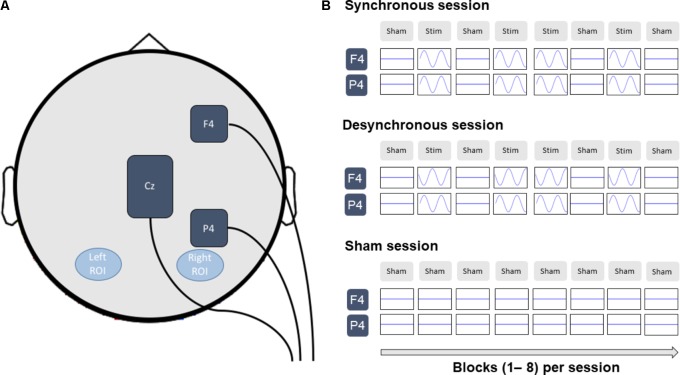
**(A)** Stimulation electrodes were placed at F4 and P4 and were stimulated synchronous or desynchronous at 10 Hz. In the synchronous stimulation condition, current flowed back via a third return electrode placed at Cz. The left and right regions of interest (ROI) contained the electrodes PO7/PO3/O1 and PO8/PO4/O2 respectively and were used for the analysis of posterior alpha power and fronto-parietal connectivity. **(B)** The experimental design consisted of alternating blocks of sham (Sham) and real (Stim) stimulation. (In the sham condition participants received sham stimulation on every block.) Only data from the last 3 sham blocks was used for the EEG analysis to avoid stimulation artifacts. For consistency, the same blocks were analyzed for the sham stimulation condition.

Stimulation was applied using a DC-Stimulator Plus device (Neuroconn) that was controlled manually. Our stimulation protocol was aimed at increasing alpha coherence between right frontal and parietal cortex. In line with our previous study (van Schouwenburg et al., 2017), we placed stimulation electrodes at the EEG 10/20 system locations F4 and P4. Both electrodes consisted of rubber patches, each with a surface area of 3 cm × 3 cm. We used a splitter to divide the current over the two electrodes placed at F4 and P4 in the synchronous stimulation condition. In this condition, we also placed a third rubber electrode, with a surface area of 5 cm × 7 cm, over Cz as a return electrode (**Figure 2A**). This site is often chosen as a return electrode (see for example Polanía et al., 2012; Neuling et al., 2013; Ruhnau et al., 2016). Electrodes were kept in place with Ten20 conductive paste.

In each stimulation condition, the electrodes at F4 and P4 received a sinusoidal alternating current with 1000 μA peak amplitude (2000 μA peak-to-peak/current density of 0.11 mA/cm^2^) at a frequency of 10 Hz. In the desynchronous condition, the current flowed back and forth between the electrodes at F4 and P4, resulting in a 180° relative phase difference between the F4 and P4 electrodes. In this condition, the third electrode was placed at Cz, but not connected to the stimulator. In the synchronous condition, the current flowed back and forth between the electrodes at F4/P4 and the electrode at Cz, resulting in a 0° relative phase difference between the F4 and P4 electrodes. The return electrode at Cz received a sinusoidal alternating current with 2000 μA peak amplitude (4000 μA peak-to-peak/current density of 0.06 mA/cm^2^) and oscillated with a 180° phase offset compared to the F4/P4 electrodes. The larger size of the Cz electrode and therefore lower current density was chosen to reduce the effects of stimulation on this site of non-interest. In the sham condition, the electrodes were wired the same as in the synchronous condition.

As described above, participants completed 8 blocks of the spatial attention task. In each block, current was ramped up to the maximum strength over 10 s. In sham stimulation blocks, current was immediately ramped down again over 10 s, while in real stimulation blocks, the current was maintained for the duration of the whole block (∼5 min) before it was ramped down over 10 s. In the sham condition, participants only received sham stimulation, while in the synchronous and desynchronous stimulation condition participants received alternating blocks of real stimulation and sham stimulation (cf. van Schouwenburg et al., 2017). Specifically, real stimulation was delivered during the second, fourth, fifth, and seventh blocks (**Figure 2B**). This allowed us to record EEG data without tACS artifacts during the remaining blocks. Because there was no difference between the stimulation conditions in terms of stimulation applied until the second block (i.e., in all stimulation conditions participants received sham during the first block), EEG data analyses focused on blocks 2–8. Specifically, only data from the last 3 sham blocks was used for the EEG analysis to avoid stimulation artifacts. For consistency, the same blocks were analyzed for the sham stimulation condition.

### Analysis of Side Effects

We tested for effect of stimulation on side effects in two different analyses. First, the scores on the visual analog scales were combined to form three factors (calmness, contentedness and alertness) (Bond and Lader, 1974) and for each factor we ran a repeated-measures ANOVA with the within-subject factors of time (pre-stimulation and post-stimulation), and stimulation condition (sham, synchronous or desynchronous). Second, self-reported side effects were compared between the three stimulation conditions using a Friedman test. Results are reported without correction for multiple comparison to ensure we do not overlook any effects of stimulation.

### Behavioral Analysis

For each stimulation condition (sham, synchronous or desynchronous), trials were divided in 6 different conditions, based on the attention condition (valid cue, neutral cue, or invalid cue), and target presentation side (left or right). Next, for each condition, we calculated accuracy and the mean RT over all trials to maximize power, separately for each subject and stimulation condition (number of trials per condition: valid-left 224; valid-right 224; neutral-left 104; neutral-right 104; invalid-left 72; invalid-right 72). Because we found a significant difference in the self-reported side effect ‘burning sensation’ between the different stimulation conditions (see Results), we analyzed the behavioral data using a linear mixed model (SPSS), allowing us to include this side effect per stimulation condition as a covariate. The model tested for main effects of stimulation condition, attention condition and target side. In addition, it tested for a main effect of burning sensation. The model also included the following interactions: stimulation condition × attention condition, stimulation condition × target side, and stimulation condition × attention condition × target side.

### EEG Data Acquisition and Preprocessing

EEG data were recorded at 512 Hz using a BioSemi ActiveTwo 64 Ag–AgCl channel setup (BioSemi, Amsterdam, Netherlands) placed according to the international 10–10 system. Note that no data could be recorded from sites where the stimulation electrodes were located. For most recording sessions, this included data from channels F4, F6, P4, P6, Cz, and CPz. Four external electrodes recorded the electro-oculogram (EOG) from vertical (below and above the left eye) and horizontal (next to the left and right outer canthi) ocular sites. Two additional electrodes were placed on both earlobes. Raw EEG data were analyzed using Fieldtrip (Oostenveld et al., 2011). Like for the behavioral analysis, we tried to replicate the analysis pipeline of our previous study as closely as possible. Only data from the sham blocks were analyzed, matched across stimulation conditions, to prevent any potential confound introduced by tACS artifacts from influencing our results. Data were high-pass filtered at 0.5 Hz, rereferenced to the earlobes and epoched from 1500 ms pre-cue to 2500 ms post-target. Epochs were demeaned/detrended and visually inspected for artifacts. Trials that contained large muscle and non-physiological artifacts were removed. The remaining trials were submitted to an independent component analysis and components containing eye blinks and/or other artifacts that clearly did not stem from physiological signal were removed. Frequency composition, event-related potentials and individual trials of each component were carefully inspected for this purpose. [Mean number of trials per condition (mean, range) across subjects and stimulation conditions: left cue (106, 92–111); neutral cue (75, 62–78); right cue (106, 89–111), no significant difference between stimulation conditions (all *p*’s > 0.28).]

We had instructed participants to keep central fixation during the experiment. To ensure that they adhered to these instructions, we checked if there were any participants that systematically used the cue to overtly direct their attention. To this end, we calculated the horizontal EOG (hEOG), by substracting the left horizontal EOG electrode from the right horizontal EOG electrode. After applying a low-pass filter of 20 Hz, we extracted the hEOG during the cue-target interval for each trial, separately for the ‘left cue’ and ‘right cue’ condition. For each subject and session, we then tested if the hEOG was significantly larger for ‘right cue’ trials compared to ‘left cue’ trials at any time point during the cue-target interval, indicating participants moved their eyes to the right side of the screen for ‘right cue’ trials and/or moved their eyes to the left side of the screen for the ‘left cue’ trials (one-sided two-sample *t*-test, Bonferroni corrected for multiple comparisons). This was the case for one or more sessions in three subjects. After exclusion of these three participants, there was no significant difference in the hEOG between ‘cue left’ and ‘cue right’ trials across participants and sessions during any of the time points of the cue-target interval (paired *t*-test Bonferroni corrected for multiple comparisons). Importantly, when we averaged over the time points of the cue-target interval, we found no main effect of cue (left vs. right) or stimulation condition (sham, synchronous or desynchronous) or interaction between the two in a repeated measures ANOVA. This indicates that fixation was not affected by stimulation. For completeness we report the average hEOG across participants for each stimulation condition and cue. Note that the values represent the voltage difference between the left horizontal EOG electrode and the right horizontal EOG electrode (right – left), such that a positive value indicate a rightward deviation from fixation and a negative value indicated a leftward deviation from fixation. (sham – left cue: 0.41; sham – right cue: 0.12; synchronous – left cue: −0.03; synchronous – right cue: −0.01; desynchronous – left cue: 0.18; desynchronous – right cue: 0.04).

### EEG Power Analysis

To test our hypothesis that tACS would affect posterior alpha activity, EEG data were submitted to a time-frequency analysis. Data from the cue-target interval were analyzed using a fixed 200 ms sliding time window moving in steps of 50 ms (cf. van Schouwenburg et al., 2017). The data in each time window were multiplied with a Hanning taper and Fourier Transformed to give the spectral power at each latency. Power values were averaged over a time window from −400 to −100 ms pretarget and across a frequency range of 8–12 Hz (width of frequency bins: 1 Hz). This time window was chosen because effects of attention need time to build up and are usually strongest toward the end of the cue-target interval. In addition, excluding the final 100 ms of the cue-target interval avoided contamination of the data by target-evoked responses.

For statistical analysis, data were averaged over trials for each attention condition separately (neutral cue, left cue, right cue) and electrodes, separately for three left parietal-occipital electrodes of interest (PO7/PO3/O1) and three right parietal-occipital electrodes of interest (PO8/PO4/O2) (cf. van Schouwenburg et al., 2017). Averaged data were then submitted to a repeated-measures ANOVA with the within-participants factors of attention condition (left cue, neutral cue, or right cue), hemisphere (left or right) and stimulation condition (sham, synchronous or desynchronous).

To show that we reproduce the main effects of spatial attention found in previous studies, we also calculated a normalized measure of alpha power with respect to the attended hemifield (Thut et al., 2006; Händel et al., 2011). The alpha modulation index was calculated for each electrode using the following formula:

$$\text{alpha modulation index}=\frac{{\text{power}}_{\text{cue left}}-{\text{power}}_{\text{cue right}}}{{\text{power}}_{\text{cue left}}+{\text{power}}_{\text{cue right}}}$$

This alpha modulation index offers a measure of oscillatory activity as a function of spatial attention and is positive if alpha power is higher on ‘cue left’ trials compared to ‘cue right’ trials and negative when alpha power is higher on ‘cue right’ trials compared to ‘cue left’ trials. Based on previous findings, we anticipated a positive alpha modulation index for the left parietal-occipital cortex where alpha power should be relatively high on ‘cue left’ trials to suppress information in the right hemifield, and relatively low on ‘cue right’ trials to allow processing of relevant information in the left hemifield. In contrast, we expected a negative alpha modulation index for the right parietal-occipital cortex where alpha power should be relatively low on ‘cue left’ trials to allow processing of relevant information in the left hemifield, and relatively high on ‘cue right’ trials to suppress information in the left hemifield (Worden et al., 2000; Händel et al., 2011; ter Huurne et al., 2013). Data were averaged over participants, time (-400 to -100 ms target-locked) and frequencies (8–12 Hz) for topographical representation.

### EEG Phase Coherence Analysis

The aim of our brain stimulation protocol was to modulate fronto-parietal synchronization of alpha activity. To explore if our stimulation protocol was successful, we calculated phase coherence. In line with our previous study, we calculated phase-locking values (PLVs) from the individual trial data from our time-frequency analysis (Lachaux et al., 1999). PLVs were calculated for each condition separately using Fieldtrip and averaged over frequencies (8–12 Hz) and time (−400 ms to −100 ms target-locked). Next, we investigated hemispheric-specific effects of stimulation on fronto-posterior connectivity. Our frontal stimulation electrode was positioned over F4. We were thus not able to record data from this electrode, and in most sessions F6 was also blocked by the stimulation electrode. Therefore, like in our previous study (van Schouwenburg et al., 2017), we assessed connectivity between F8 and our left and right parietal-occipital electrodes of interest, assuming that given the high conductivity of the scalp, we would still be sensitive to picking up effects of tACS at nearby sites. PLVs were averaged for the clusters of left and right electrodes separately. Data were submitted to a repeated-measures ANOVA with the within-participants factors of attention condition (left cue, neutral cue, or right cue), hemisphere (left or right) and stimulation condition (sham, synchronous, or desynchronous).

Again, an additional analysis was performed to show that we reproduce common findings in the spatial attention literature. We calculated PLVs over the same time and frequency windows but now between a left frontal cluster and left parietal cluster, and a right frontal cluster and right parietal cluster [conform for example (Sauseng et al., 2011)]. As described above, F4 and P4 were blocked by the stimulation electrode. Therefore, for each cluster, we selected the four electrodes surrounding the site of stimulation (left frontal: F1, AF3, F5, FC3; left parietal: PO3, P1, P5, CP3; right frontal: F2, AF4, F6, FC4; right parietal: PO4, P2, P6, CP4). In many sessions some of these (right-sided) electrodes were blocked by the stimulation electrode as well (usually F6 and P6). If this was the case, these electrodes were not included in the analysis, and for consistency we also excluded the left-sided equivalent of that electrode for the analysis of that particular session. Again, data were averaged over the cluster of electrodes and trials, and were submitted to a repeated-measures ANOVA with the within-participants factors of attention condition (left cue, neutral cue, or right cue), hemisphere (left or right) and stimulation condition (sham, synchronous, or desynchronous).

## Results

### Side Effects

Stimulation did not affect subjective feelings, as indicated by the fact that none of the factors of the subjective feelings ratings showed a stimulation condition × time interaction [calmness, *F*(2,38) = 0.419, *p* = 0.661; contentedness, *F*(2,38) = 0.059, *p* = 0.943; alertness, *F*(2,38) = 0.651, *p* = 0.527]. (Note that there were also no main effects of time, or stimulation condition on any of the factors.)

However, looking at the self-reported side effects, we did find a significant effect of stimulation condition. Specifically, there was a main effect of stimulation condition on the amount of burning sensation [χ^2^(20) = 13.650, *p* = 0.001] reported after the stimulation. This was due to a lower amount of burning sensation during the desynchronous condition, compared to the synchronous and sham condition (mean rank: synchronous, 2.28; desynchronous, 1.53: sham, 2.20).

### Behavioral Results

**FIGURE 3:**
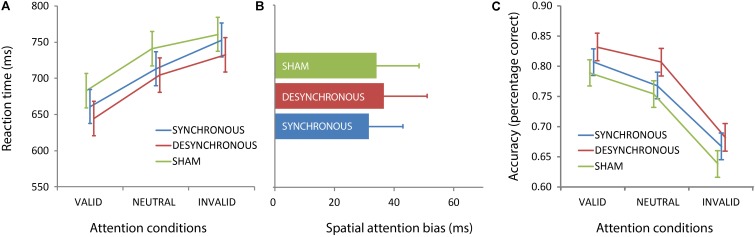
Attention modulates performance, independent of fronto-parietal transcranial alternating current stimulation (tACS). Mean reaction times **(A)** and accuracy levels **(C)** are plotted for each attention condition and stimulation condition (mean ± standard error), collapsed over stimuli in the left and right hemifield. Both reaction times and accuracy show a main effect of attention condition, reflecting better performance to validly cued (attended) targets compared to neutrally and/or invalidly cued targets. This pattern was not affected by tACS, as reflected in the absence of an interaction between stimulation condition and attention condition. Overall, reaction times were faster during active compared to sham tACS, as captured by a significant main effect of stimulation condition **(A)**. For accuracy we also found a main effect of stimulation, which was driven by better performance in the desynchronous condition **(C)**. The absence of a stimulation condition by target side interaction is represented in **(B)**, which shows the spatial attention bias (average reaction times for right hemifield stimuli minus average reaction times for left hemifield stimuli) for the three different stimulation conditions. Error bars represent standard error of the mean.

As described in the methods section, we used a linear mixed model to control for differences in the amount of burning sensation between the stimulation conditions. For RT, we found a main effect of attention condition [*F*(2,321) = 46.583, *p* < 0.001] (**Figure 3A**). In line with many other studies, participants were faster in valid trials, followed by neutral trials, and invalid trials. (Results from *post hoc* pairwise comparisons: valid vs. neutral, *p* < 0.001; valid vs. invalid, *p* < 0.001; neutral vs. invalid, *p* = 0.001.) In our previous study, we found that participants who received sham stimulation were faster for targets in the right hemifield, while participants who received synchronous stimulation showed no spatial attention bias. This stimulation condition × target side interaction was not replicated in the current study [*F*(2,321) = 0.038, *p* = 0.962]. Instead, we found that participants were generally faster for targets in the right hemifield, as reflected by a main effect of side across stimulation conditions [main effect of side: *F*(1,321) = 21.252, *p* < 0.001] (**Figure 3B**). No other differential effects of stimulation condition were observed. Instead, both synchronous and desynchronous stimulation speeded responses across task conditions [main effect of stimulation; *F*(2,332) = 5.821, *p* = 0.003] (**Figure 3A**). *Post hoc* pairwise comparisons showed that this effect was driven by significantly faster response times in the desynchronous compared to sham condition (*p* = 0.001), and significantly faster response times in the synchronous vs. sham condition (*p* = 0.035). We also found a main effect of burning sensation [*F*(1,313) = 12.508, *p* < 0.001], suggesting that RT was correlated with the amount of burning sensation.

For accuracy, we found a main effect of condition [*F*(2,322) = 75.178, *p* < 0.001], and a main effect of side [*F*(1,322) = 35.506, *p* < 0.001] (**Figure 3C**). The results followed the same pattern as for the RTs; participants were most accurate on valid trials, followed by neutral trials, and were least accurate on invalid trials. (Results from *post hoc* pairwise comparisons: valid vs. neutral, *p* = 0.009; valid vs. invalid, *p* < 0.001; neutral vs. invalid, *p* < 0.001.) Also, participants were more accurate on targets that were presented in the right hemifield (main effect of side; *F*(1,322) = 35.506, *p* < 0.001). Similar to the results from the RT analyses, we found a main effect of stimulation condition [*F*(2,338) = 338.324, *p* = 0.004]. Participants were significantly more accurate in the desynchronous compared to sham condition (*p* = 0.001), while the accuracy levels between the other conditions did not differ significantly (synchronous vs. desynchronous: *p* = 0.065; synchronous vs. sham: *p* = 0.109).

### Alpha Power

**FIGURE 4:**
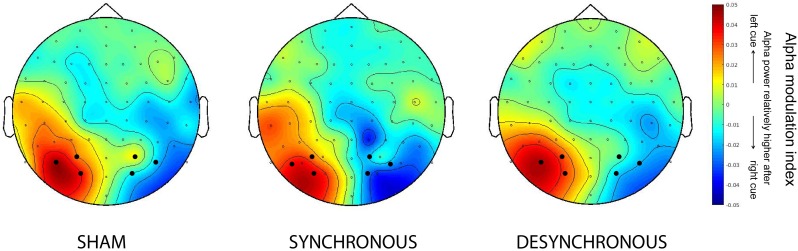
Spatial attention modulated prestimulus alpha lateralization, but fronto-parietal tACS did not affect this top–down attention effect. Topographical representations of the alpha modulation index averaged over time (−0.4 s to −0.1 s target-locked), and frequencies (8–12 Hz) are shown separately for the three different stimulation conditions. In each stimulation condition, alpha modulation (ipsilateral increase and contralateral decrease in alpha power relative to the attended direction) is present for both hemispheres.

Our first EEG analysis focused on effects of tACS on prestimulus alpha power over posterior electrodes as a marker of top-down spatial attention. Mirroring the behavioral findings, no differential effect of stimulation condition on posterior alpha was observed: An ANOVA on raw alpha power showed a main effect of attention condition [*F*(2,38) = 4.606, *p* = 0.016], and a hemisphere × attention condition interaction [*F*(2,38) = 4.555, *p* = 0.041], but no main effect of stimulation condition or interactions between stimulation condition and any of the other factors. (The same results were obtained when looking at 10 Hz only, the frequency of our tACS stimulation, instead of a 8–12 Hz frequency band: Main effect of attention *F*(2,38) = 4.542, *p* = 0.017; hemisphere × attention condition interaction *F*(1,21) = 4.505, *p* = 0.042.) These findings replicate common reports of a spatial attention-induced increase in prestimulus alpha power over ipsi- compared to contralateral posterior scalp regions; *post hoc* pairwise comparisons showed that the main effect of attention was caused by an increase in posterior alpha power in the cued attention conditions (left cue and right cue) compared to the neutral attention condition. (left cue vs. neutral cue, *p* = 0.027, right cue vs. neutral cue, *p* = 0.014; left cue vs. right cue, *p* = 0.409). When further splitting the data as a function of hemisphere, the following pattern emerged: during the neutral attention condition alpha power was relatively low in both the left and right hemisphere, while for the left and right cued conditions, alpha power was modulated as a function of the cued hemifield in a hemispheric-specific manner. In the left cue condition, alpha was relatively low over the right (contralateral) hemisphere, while relatively high over the left (ipsilateral) hemisphere. The opposite was true for the right cue condition. This is in line with the idea that alpha power decreases contralateral to allow processing of task-relevant information, while it increases ipsilateral to inhibit processing of task-irrelevant information. The attention by hemisphere interaction can be nicely illustrated by means of a modulation index (see Materials and Methods). **Figure 4** shows that the modulation index is positive for the left posterior cortex, but negative for the right posterior cortex, in line with many previous studies. This confirms that participants were directing their attention to the cued hemifield. However, it also confirms that there was no difference between the three stimulation conditions.

### Alpha Phase Coherence

**FIGURE 5:**
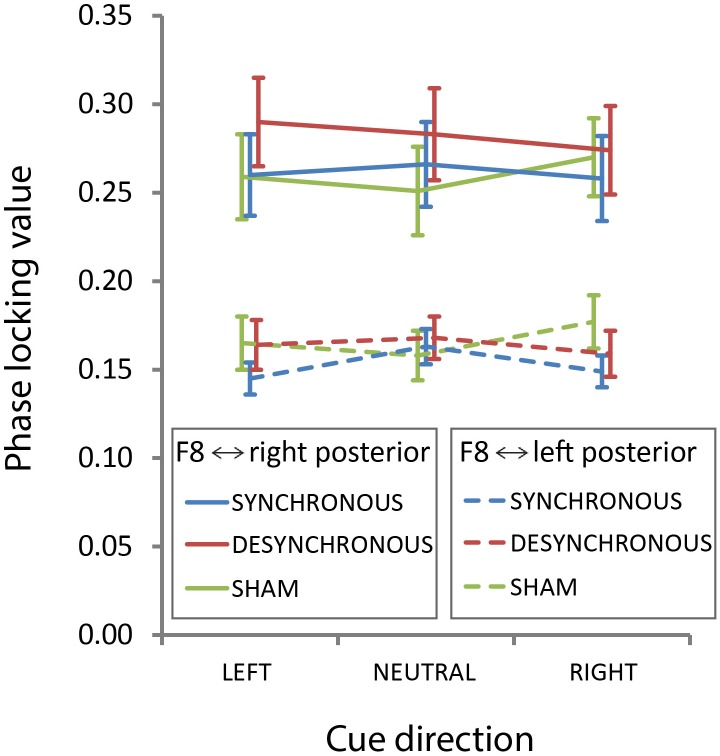
Transcranial alternating current stimulation effects on fronto-posterior connectivity. Phase-locking values from F8 to our left and right electrodes of interest averaged over time (−0.4 s to −0.1 s target-locked), and frequencies (8–12 Hz) are shown here separately for each stimulation condition and attention condition. Error bars represent standard error of the mean.

To test if stimulation affected fronto-posterior connectivity, next, we calculated PLVs between F8 and our left and right posterior electrodes of interest, as such replicating the analysis from our previous study (van Schouwenburg et al., 2017). We found a main effect of hemisphere [*F*(1,19) = 118.653, *p* < 0.001], such that overall coherence was higher between F8 and the right posterior electrodes compared to the coherence between F8 and left posterior electrodes (**Figure 5**). (Note that this effect was in the opposite direction in our previous study, where we found higher coherence between F8 and left posterior electrodes.) In addition, the full three-way ANOVA showed a significant interaction between attention condition and stimulation condition [*F*(4,76) = 3.645, *p* = 0.009]. This interaction was driven by a main effect of attention condition that just reached significance in the sham stimulation condition [*F*(2,38) = 3.260, *p* = 0.049], but not in the synchronous [*F*(2,38) = 2.014, *p* = 0.147] and desynchronous [*F*(2,38) = 1.139, *p* = 0.331] condition (**Figure 5**). These findings are contrary to what one would expect if synchronous stimulation improves fronto-parietal communication, as they indicate that active stimulation, including synchronous tACS, may have eliminated attention-induced increases in fronto-parietal connectivity. Also, we do not replicate the hemisphere by stimulation condition interaction that we found in our previous study [*F*(2,38) = 0.469, *p* = 0.629]. When we only looked at 10 Hz (our stimulation frequency) we found a main effect of hemisphere [*F*(1,19) = 101.317, *p* < 0.001], but the attention condition by stimulation condition interaction did not reach significance [*F*(4,76) = 2.408, *p* = 0.057].

In a supplementary analysis, we aimed to show that we reproduce commonly found effects of attention on fronto-posterior connectivity. We calculated PLVs between a cluster of frontal and parietal electrodes (surrounding F3/F4 and P3/P4) in both the left and right hemisphere. Connectivity was stronger in the right hemisphere [main effect of hemisphere; *F*(1,19) = 111.666, *p* < 0.001] and on trials in which participants were cued to the right hemifield [main effect of attention condition; *F*(2,38) = 4.137, *p* = 0.024]. Importantly, in line with previous findings, we also found an interaction between hemisphere by attention condition (*p* = 0.002). In the left hemisphere, connectivity was significantly stronger following a cue to the right hemifield, compared to a left cue or neutral cue. (right cue vs. left cue, *p* = 0.002, right cue vs. neutral cue, *p* = 0.017, left cue vs. neutral cue, *p* = 0.199). In the right hemisphere, connectivity was strongest on left cue trials, followed by right cue trials, and weakest after a neutral cue, but there was no significant difference between the three conditions (right cue vs. left cue, *p* = 0.805, right cue vs. neutral cue, *p* = 0.134, left cue vs. neutral cue, *p* = 0.061). Again, there was no main effect of stimulation condition, or any interactions with stimulation condition (all *p*-values > 0.1).

## Discussion

Previous TMS studies have shown that the fronto-parietal attention network is causally involved in top–down attention and modulates neural activity at the level of the visual cortex (Capotosto et al., 2009; Sauseng et al., 2011; Marshall et al., 2015). It has been suggested that this network communicates through coherence in the alpha band (Sauseng et al., 2005; Zanto et al., 2010; Sadaghiani et al., 2012; Doesburg et al., 2016). We found preliminary evidence supporting this theory in a previous tACS study and in the current tACS study we aimed to conceptually replicate and extend those findings.

Previously, we showed that, compared to a group of participants that received sham stimulation, synchronous fronto-parietal alpha band stimulation of the right hemisphere altered alpha coherence between the frontal and parietal-occipital cortex in a hemisphere-specific manner. Behaviorally, the groups showed a significant difference in terms of a spatial attention bias. Based on these findings, we concluded that alpha coherence within the fronto-parietal network subserves top–down control of attention. However, in the current study, we do not replicate these findings, although typical effects of spatial attention on behavior (cue validity effect) and neural activity (pre-stimulus alpha asymmetry) were observed. All our measures of attention (i.e., the spatial bias in behavioral performance, posterior alpha power and fronto-parietal connectivity) were not differentially affected by synchronous versus desynchronous fronto-parietal alpha tACS.

These results are unexpected, also since the currently employed within-subject design was anticipated to be more sensitive to tACS effects. Yet, our study was not an exact replication of van Schouwenburg et al. (2017), and several differences in stimulation parameters and experimental task between the two studies may explain the discrepancy in findings. In terms of the stimulation, the current density at F4 and P4 was threefold higher in the previous study compared to the current study (0.32 mA/cm^2^ vs. 0.11 mA/cm^2^). This was due to differences in the size of the stimulation electrodes: the applied current (2000 μA peak-to-peak) was the same in the two studies. Perhaps the current density in the current study was not sufficient to modulate coherence between frontal and parietal cortex. Indeed, (after-) effects of stimulation seem to increase with increasing current density (Nitsche and Paulus, 2000). Nevertheless, previous studies using similar current densities (or lower) as in the current study have reported significant effects of electrical stimulation on performance (see for example Polanía et al., 2012; Jaušovec et al., 2013; Hopfinger et al., 2016). Another difference between the two studies is that, in our previous study, we stimulated participants at their individual alpha frequency, while in the present study we stimulated everyone at the same frequency (10 Hz). This frequency was chosen because it was closest to the average frequency of stimulation in our previous study (10.3 Hz). A recent *in vitro* study showed that weak electric fields could enhance endogenous oscillations, but only when the stimulation frequency matched the intrinsic frequency (Schmidt et al., 2014). Similarly, using rTMS, Romei et al. (2016) found that effects of stimulation were strongest when the stimulation frequency was consistent with the individual peak frequency. These findings suggest that matching the stimulation frequency to the endogenous frequency of a given individual might be essential for the effectiveness of the stimulation. It should be noted, however, that other studies using similar stimulation parameters as in the present study did find effects, albeit in the domain of working memory rather than spatial attention (Polanía et al., 2012; Violante et al., 2017). Finally, the return electrodes were placed at different locations in the two studies (C2/C4/C6 vs. Cz), which could have led to different current flow patterns at the level of the brain. In addition, while it is often assumed that the strongest stimulation takes place directly under the electrodes, modeling studies suggest that the peak electrical field may occur *between* the electrodes (Datta et al., 2009; Miranda et al., 2013; Opitz et al., 2015). As a consequence, the peak of stimulation might have been at different sites in the two studies.

In addition to changes in the stimulation parameters, we also adapted the experimental paradigm in the present study. First, whereas in our previous study, the cue was 100% valid, in the current study, on some trials the target appeared in the hemifield opposite to the cued hemifield, reducing cue validity to 75%. This allowed us to determine if fronto-parietal tACS may selectively strengthen top–down attention, or may also affect bottom–up attention (as reflected by changes in attentional reorienting on invalid compared to neutral trials). Second, in the current study on each trial a distractor was presented in the hemifield opposite to the target location, while no distractors were present in the previous study. We reasoned that since occipital alpha power is theoretically associated with suppression of irrelevant information, stimulation effects might surface particularly in the presence of such irrelevant information. These changes in experimental design may have also influenced our ability to observe tACS effects, if these are more pronounced in more simple tasks that only require directing of spatial attention.

It is important to emphasize that we did replicate common findings in the spatial attention literature, both in terms of behavior as well as neural activity (Posner, 1980; Worden et al., 2000; Sauseng et al., 2005). That is, subjects were fastest on validly cued trials and slowest on invalidly cued trials. Moreover, spatial attention was associated with changes in pre-stimulus alpha asymmetry, indicative of a top–down spatial attentional bias. It is therefore unlikely that the absence of stimulation effects in the current study are due to the specific experimental paradigm used or particular parameters of the EEG analysis.

Naturally, we should also consider the possibility that the results from our previous study were a spurious finding. Indeed, findings from non-invasive brain stimulation studies often fail to replicate (Veniero et al., 2017; for review see Reteig et al., 2017), casting doubt on the effectiveness of this technique (Horvath et al., 2015; Medina and Cason, 2017).

The goal of our study was to investigate if increasing coherence with synchronous stimulation of frontal and parietal cortex would alter spatial attention. The desynchronous stimulation condition was added as a control condition to control for frontal and parietal stimulation in of itself. Although this approach has been used previously (Polanía et al., 2012), a recent study challenged the validity of this particular design (Saturnino et al., 2017). In this study, using electrical field simulations, it was shown that during the synchronous stimulation condition, the strongest polarization takes place under the return electrode. This is due to the fact that the current strength at the return electrode is twice that of the current strength at the active electrodes. In the desynchronous stimulation condition, the strongest polarization takes places under the active electrodes. Besides a difference in relative phase, the two stimulation conditions therefore also differ in terms of peak field strength and focality and consequently the desynchronous stimulation constitutes a poor control condition. The study also suggests alternative electrode setups that are better suited to test the effect of phase synchrony using tACS. Future studies should follow these recommendations.

Nevertheless, evidence for the notion that the fronto-parietal network exerts top–down control over visual processing through communication in the alpha band is accumulating. Previous studies have shown that alpha coherence between frontal cortex and parietal(-occipital) cortex is modulated as a function of attention (Sauseng et al., 2005; Zanto et al., 2010; Doesburg et al., 2016). Moreover, blood oxygen level dependent (BOLD) activity in the fronto-parietal network correlates with alpha power (Sadaghiani et al., 2012). Findings from both animal and human studies furthermore indicate that bottom–up communication takes place in the gamma range and top–down communication happens through coherence in the alpha/beta range (Phillips et al., 2013; van Kerkoerle et al., 2014; Bastos et al., 2015; Michalareas et al., 2016; Scheeringa and Fries, 2017; Lobier et al., 2018). Causal evidence for top–down communication in the alpha band comes from a study in which TMS was used to perturb neural activity in the inferior frontal junction, part of the fronto-parietal network, which led to a reduction in attentional modulation of alpha band coherence between the frontal and parietal-occipital cortex (Zanto et al., 2011). Yet, whether electrical stimulation can be used to enhance fronto-parietal interactions and attentional performance thus remains unclear (see also Reteig et al., 2017). We should note that we did observe an effect of stimulation on attentional-induced changes in prestimulus fronto-posterior alpha band coherence. That is, while fronto-posterior connectivity was higher after an attention-directing vs. neutral cue in the sham stimulation condition, this attention-related increase in fronto-posterior connectivity was not observed during synchronous or desynchronous tACS. Yet, this effect is in the opposite direction of what one would expect if synchronous stimulation improves fronto-parietal communication, and was not observed in our previous study. Further, the effect was similar for synchronous and desynchronous tACS, suggesting that it may not be related to changes in phase coherence *per se*, although we cannot claim this with certainty, since in our design these two conditions differed also in other aspects than phase (Saturnino et al., 2017). Future studies are thus necessary to determine the reliability of this effect.

In our study we found that participants were overall faster for targets that appeared in the right hemifield. The same was true in our previous study for participants that only received sham stimulation. In that study, participants responded with their right hand, which might have induced a Simon effect. However, in this study we asked participants to respond bimanually to exclude this potential confound, and yet, we still find this rightward bias in terms of response times. We could speculate that perhaps the fact that stimulation was applied to the right side of the head might have attracted people’s attention to that side of space. However, additional research with left sided stimulation is needed to confirm this. Behaviorally, we also found that F4/P4 stimulation (regardless of relative phase) decreased RTs in all attention conditions. This finding could reflect an unanticipated, but true effect of stimulation on processes other than attention, such as motor preparation. Alternatively, the general speeding up of response speed in both stimulation conditions could reflect non-specific effects related to active stimulation, such as a general effect on alertness.

In sum, current evidence for a causal role of alpha band coherence in fronto-parietal top–down control in spatial attention is inconclusive. A direct replication study, using the same stimulation parameters and experimental design, is necessary to determine if our previous results (van Schouwenburg et al., 2017) were false positive findings. More research is also necessary that systematically examines the factors that may influence the effectiveness of transcranial alternating current stimulation, including current density, placement of the electrodes and specific frequency of stimulation (individualized or not). This will hopefully help determine whether cognitive functions, like attention, can be improved through synchronization of oscillatory activity between brain regions. This knowledge does not only have scientific relevance, but also important clinical implications.

## Author Contributions

MvS and HS conceived the study. All authors contributed to the design of the study. LS, RdK, and MvS acquired the data. LS, RdK, and MvS analyzed the data. MvS and HS wrote the paper with help from all authors.

## Conflict of Interest Statement

The authors declare that the research was conducted in the absence of any commercial or financial relationships that could be construed as a potential conflict of interest.
